# Exosomes function in antigen presentation during an *in vivo Mycobacterium tuberculosis* infection

**DOI:** 10.1038/srep43578

**Published:** 2017-03-06

**Authors:** Victoria L. Smith, Yong Cheng, Barry R. Bryant, Jeffrey S. Schorey

**Affiliations:** 1 Department of Biological Sciences, Eck Institute for Global Health, University of Notre Dame, Notre Dame, Indiana 46556, USA

## Abstract

*Mycobacterium tuberculosis*-infected macrophages and dendritic cells are limited in their ability to present antigen to CD4+ T cells suggesting that other mechanism of antigen presentation are driving the robust T cell response observed during an *M. tuberculosis* infection. These mechanisms could include antigens present in apoptotic bodies, necrotic debris, exosomes or even release of non-vesicular antigen from infected cells. However, there is limited data to support any of these mechanisms as important in driving T cell activation *in vivo*. In the present study we use Rab27a-deficient mice which show diminished trafficking of mycobacterial components to exosomes as well as *M. tuberculosis* strains that express recombinant proteins which traffic or fail to traffic to exosomes. We observed that exosomes released during a mouse *M. tuberculosis* infection contribute significantly to its T cell response. These finding imply that exosomes function to promote T cell immunity during a bacterial infection and are an important source of extracellular antigen.

Studies of animal models and tuberculosis (TB) patients indicate that a host mounts a robust T cell response to an *M. tuberculosis (Mtb*) infection and this response is essential for controlling the infection^1^. However, the mechanism(s) by which antigens are presented to T cells is still unclear. Possible mechanisms include direct antigen presentation by infected cells as well as uptake of necrotic cells or apoptotic bodies caring mycobacterial proteins. Recent studies suggest that “free” antigen can be released from infected cells and promote cross-priming^2^. Our published data also suggest that exosomes could play a role in *Mtb* antigen presentation^3^,^4^. Exosomes are membrane-bound vesicles of 30–150 nm in size that are released when multivesicular bodies (MVB) fuse with the plasma membrane releasing their intraluminal vesicles into the extracellular environment^5^,^6^. Exosomes generally function in intracellular communication and have been implicated in many physiological and pathological processes^7^. The release of exosomes has been implicated as a mechanism through which components derived from intracellular pathogens gain access to the immune system. Previous publications support this possibility as exosomes released from cells infected with intracellular pathogens such as *Salmonella, Toxoplasma gondii*, and *M. tuberculosis*, to name a few, contain pathogen components^8^. Furthermore, our earlier studies demonstrate that mice vaccinated with exosomes containing mycobacterial antigens can activate both CD4+ and CD8+ T cells and can protect these mice against infection to an extent comparable to *M. bovis* BCG^3^. Nevertheless, there is limited data to support any of the antigen presentation mechanisms as important in driving T cell activation *in vivo* and recent studies suggest that release of non-vesicular antigen from infected cells may in fact limit the T cell response^9^. Our inability to define the most relevant mechanisms of antigen presentation during the course of an *Mtb* infection stem, in part, from a lack of *in vivo* models where exosomes and other biological processes involved in antigen presentation can be blocked or modulated.

Previous published reports indicate that Rab27a and Rab27b may play an important role in exosome biogenesis at least in certain cell types^10^,^11^. Rab27 is a small molecular weight GTPase that is a member of the Ras GTPase superfamily. Through the use of a guanine-nucleotide dependent switch, they are known to regulate steps in membrane trafficking including: vesicle formation, vesicle trafficking, tethering, and fusion with target organelles^12^. Rab27a appears to mediate MVB docking to the plasma membrane during exosome biogenesis in FL3 and SLT4 metastatic cell lines, lung adenocarcinoma cells (A549) and the HeLa B6H4 tumor cell line^10^. Our present work extends these finding to macrophages where loss of Rab27a expression leads to diminished exosome production. These results suggest that Rab27a-deficient mice could serve as a useful model to evaluate exosome production during an infection. We found *Mtb*-infected Rab27a-deficient mice to have reduced exosome production and diminished activation of antigen-specific T cells as well as a diminished ability to control an *Mtb* infection compared to wild-type mice.

However, since Rab27a has been implicated in neutrophil degranulation as well as in other process that can impinge on immune function^13^,^14^, it was important to use additional approaches to evaluate exosomes as drivers of T cell activation during an *in vivo Mtb* infection. For this objective we generated BCG or *Mtb* H37Rv strains that expresses tagged DsRed or the mycobacterial protein HspX which differed in their trafficking to exosomes. When mice were infected with the different mycobacterial strains, increased T cell response to DsRed and HspX was observed when these proteins were targeted to exosomes. Altogether our data provides direct evidence for exosome-mediated T cell activation and suggest that cross presentation of antigen during an *in vivo Mtb* infection can be an important mechanism for eliciting an acquired immune response.

## Results

### Rab27a functions in exosome release in murine bone marrow-derived macrophages

**Figure 1:**
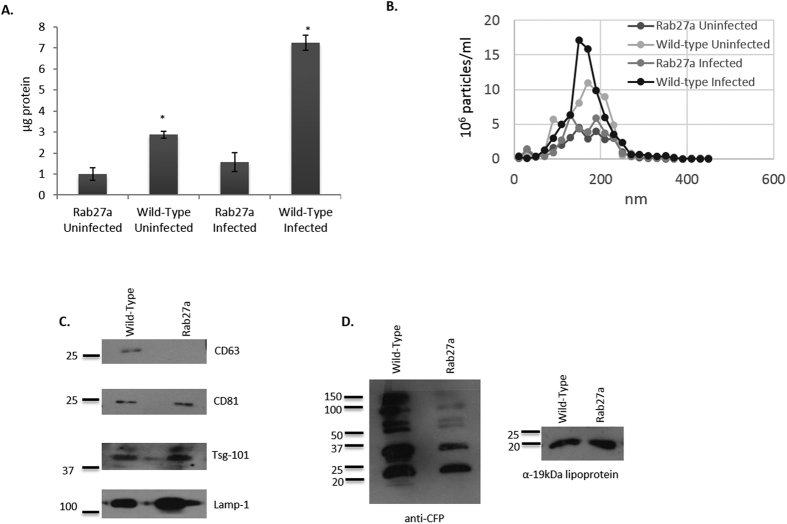
Quantitative and qualitative changes in exosomes released from *Mtb*-infected Rab27a-deficient BMMs. Exosomes were isolated from the cell culture supernatant of C57BL/6 and Rab27a-deficient BMMs infected either with *Mtb* at a 3:1 MOI or left uninfected. Purified exosomes were **(A)** quantified for protein concentration by BCA and **(B)** for vesicle number by Nanosight analysis. Shown is a representative Nanosight profile for exosomes released from infected or uninfected wild-type or Rab27a-deficient macrophages. **(C)** 10 μg of exosomes were probed for the presence of exosomal markers, Lamp-1, CD63, CD81 and Tsg-101 by western blot. **(D)** 20 μg of exosomes from *Mtb* infected BMM were assayed for mycobacterial proteins using an antibody that recognizes multiple *Mtb* culture filtrate proteins (CFP) and an antibody against the *Mtb* 19 kDa lipoprotein. The BCA data is the average protein concentration across 3 independent experiments +/−SD and statistical analysis was performed comparing Rab27a-deficient to wild-type infected macrophages (*p < 0.05). The NanoSight and western blot data are representative of three independent experiments. Uncropped western blots are shown in Supplementary Figure 1.

Rab27a had been identified in previous studies as an key regulator of MVB fusion with the plasma membrane, thereby regulating an important step in exosome biogenesis. However, the extent to which Rab27a mediates exosome release in macrophages had not been previously defined. To address this issue, bone marrow derived macrophages were isolated from Rab27a-deficient and wild-type C57BL/6 mice. Macrophages derived from Rab27a-deficient mice when infected with *Mtb* showed an 80% decrease in the number of exosomes in the culture media compared to the number of exosomes released from infected wild-type C57BL/6 macrophages (Fig. 1A,B). Furthermore, the protein markers found on exosomes released from Rab27a deficient cells may represent a unique subpopulation of exosomes whose biogenesis is mediated by alternative secretion mechanisms. As shown in Fig. 1C, the vesicles isolated from Rab27a-deficient macrophages featured a unique exosomal protein marker profile, notably a decreased CD63 expression which is consistent with previously reported studies^10^. Given that exosome secretion from Rab27a-deficient cells may represent a specific subpopulation, we sought to characterize the mycobacterial protein profile on these exosomes. Rab27a-deficient macrophages were infected with *Mtb* and probed for mycobacterial proteins using an antibody pool that was generated against *Mtb* culture filtrate proteins. We observed a general diminished presence of mycobacterial proteins but similar 19KDa lipoprotein concentration in/on exosomes secreted from *Mtb* infected Rab27a-deficient compared to wild-type macrophages (Fig. 1D). The loaded samples were normalized for protein concentration and therefore even a larger percentage of the total exosome material released from the Rab27a-infected relative to wild-type infected macrophages was used for the western blot. Importantly, we observed no difference in *Mtb* uptake or its survival between wild-type and Rab27a-deficient macrophages (Supplementary Figure 2). The diminished presence of mycobacterial proteins in exosome released from Rab27a-deficient macrophages suggest that these exosomes may have reduced immune system stimulatory activity. This prediction is supported by the observed limited TNF-α and RANTES production by macrophages treated with exosomes isolated from infected Rab27a-deficient macrophages as compared to exosomes from wild-type cells (Supplementary Figure 3).

### Rab27a-defcient mice show reduced exosomes concentrations in serum, which correlates with an increase in bacterial burden and decreased T cell activation

**Figure 2:**
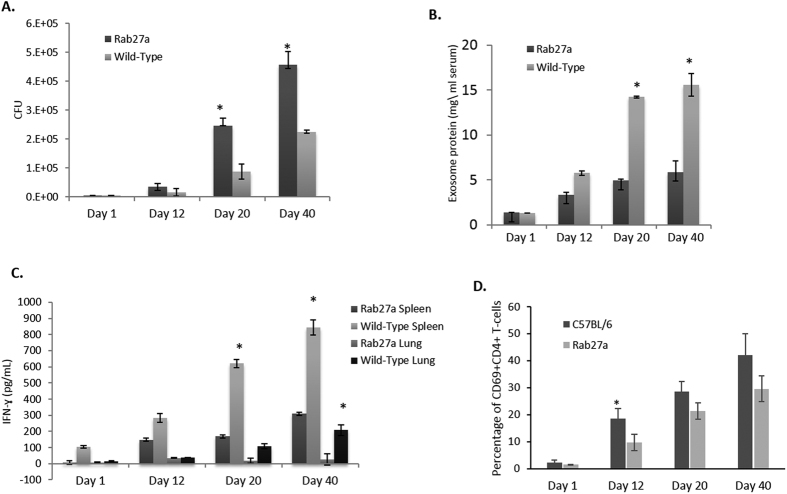
Rab27a-deficient mice infected with *Mtb* show decreased serum exosome concentration, increased bacterial load and diminished T cell response compared to WT infected mice. Bacterial load in the spleen **(A)** and serum exosome concentration **(B)** were defined at different times post-infection. **(C)** Cells isolated from lung and spleens of infected mice at the times indicated were stimulated *ex vivo* with *Mtb* whole cell lysate and the amount of secreted IFN-γ quantified by ELISA. The results are expressed as the IFN-ɣ concentration after stimulation of 1 × 10^6^ cells with the *Mtb* cell lysate. **(D)** Splenocytes were isolated from wild-type C57BL/6 and Rab27a-deficient mice at different times post-infection with *Mtb.* The cells were stained with PE-conjugated anti-mouse CD69 and CD4 or with an isotype control antibody. CD4+ T cells were analyzed for CD69 surface expression with a Beckman Coulter flow cytometer, and the percentage of CD69 positive relative to total CD4+ cells was calculated. Results are defined for each mouse with +/−SD and statistical analysis was performed comparing Rab27a-deficient mice to wild-type infected mice (*p < 0.05). The data is representative of three independent experiments for a total of 9 mice per mouse strain per time point.

Most exosome studies which define their contribution to an immune response have been done in the context of autoimmunity and cancer biology, while little has been done to elucidate their role in modulating an immune response to infectious diseases. We have previously shown that exosome production in *M. bovis* BCG-infected C57BL/6 mice correlated with bacterial load^15^. Given that Rab27a deficiency results in decreased exosome release from cells, we sought to characterize the progression of disease in Rab27a-deficient mice infected with mycobacteria. Rab27a-deficient and wild-type C57BL/6 mice were infected with 10^6^ *Mtb* or *M. bovis* BCG by retro-orbital injection and the mice were sacrificed at different times post-infection. Rab27a-deficient mice infected with *M. bovis* BCG or *Mtb* showed a marked increase in bacterial burden over time when compared to wild-type infected mice (Fig. 2A and Supplementary Figure 4) and this correlated with lower concentration of exosomes found in the serum (Fig. 2B and Supplementary Figure 4). Furthermore, compared to wild-type infected mice, cells isolated from the lung and spleen of *Mtb*-infected Rab27a-deficient mice showed diminished IFN-γ production when stimulated *ex-vivo* with *Mtb* whole cell lysate (Fig. 2C). Spleen CD4+ T-cell activation as measured by CD69 expression was also significantly diminished 12-days post infection (Fig. 2D). Histopathology of lung sections were generated to identify lymphocyte infiltration (Supplementary Figure 5). There was no significant differences in overall pathology between Rab27a-deficient and wild-type C57BL/6 mice although a general trend toward increased pathology in the Rab27a-infected mice was observed (Supplementary Figure 5). Altogether these results suggest a diminished T-cell immune response in Rab27a-deficient mice.

### Exosomes isolated from M.tb infected Rab27a-deficient mice show a reduced capacity to elicit a pro-inflammatory response

**Figure 3:**
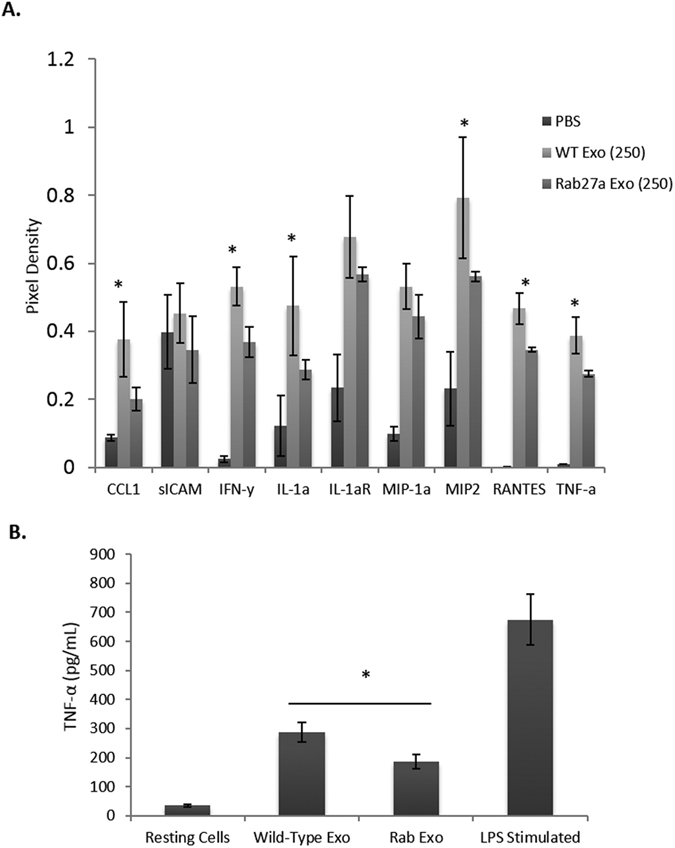
Exosomes purified from the serum of *Mtb*-infected wild-type mice were more pro-inflammatory compared to exosomes from infected Rab27a-deficient mice. Serum exosomes from *Mtb*-infected wild-type and Rab27a-deficient mice (3 mice/group) were purified and used at 250 μg/ml to treat BMMs for 16 hours. **(A)** The supernatants were harvested and assayed for specific proteins using a mouse cytokine array. The pixel densities for each spot of the array were calculated using ImageJ software and plotted. Results are defined for each individual mouse +/− SD and statistical analysis was performed comparing Rab27a-deficient mice to WT infected mice (*p < 0.05). **(B)** The culture supernatants were also analyzed for TNF-α by ELISA. The results are the mean of three separate experiments (9 mice total) with SD shown. Statistical analysis was performed comparing Rab27a-deficient to wild-type infected mice (*p < 0.05). Exo; exosomes. Rab; Rab27a.

Given our *in vitro* study which showed that exosomes derived from Rab27a-deficient macrophages have a diminished capacity to induce macrophage production of pro-inflammatory cytokines, we addressed whether this was also true for the *in vivo* derived exosomes. Exosomes were purified from serum of Rab27a-deficient and wild-type C57BL/6 infected mice and PBS-treated mice at days 10 and 20 post-infection. Pooled exosomes from these time points were used to treat BMMs. Serum-derived exosomes from wild-type and Rab27a-deficient mice were normalized to protein concentration (250 μg/mL, equal to ~2.5 × 10^10^ exosomes) and the BMMs were treated for 16 hours or left untreated. Supernatants were assayed for cytokine and chemokine levels, using a mouse cytokine array kit and pixel intensities were defined and plotted (Fig. 3A). Exosomes derived from the serum of wild-type C57BL/6 infected mice induced a significantly higher level of CCL1, IFN-γ, RANTES, MIP-2, IL1aR, and TNF-α as compared to exosomes isolated from infected Rab27a-deficient mice. To confirm the array results, BMMs were again treated with exosomes isolated from Rab27a-deficient or wild-type infected mice and the BMM supernatants were harvested 16 hours post-infection and analyzed for TNF-α concentration by ELISA. The results were comparable to the cytokine array, showing an approximate 30% reduction in TNF-α secreted from macrophages treated with exosomes isolated from infected Rab27a-deficient mice compared to infected wild-type mice (Fig. 3B). These results suggest that exosomes released from infected Rab27a-deficient mice are less pro-inflammatory.

### Macrophage infected with *M. bovis* BCG expressing Ag85A-DsRed release exosomes containing the fusion protein

**Figure 4:**
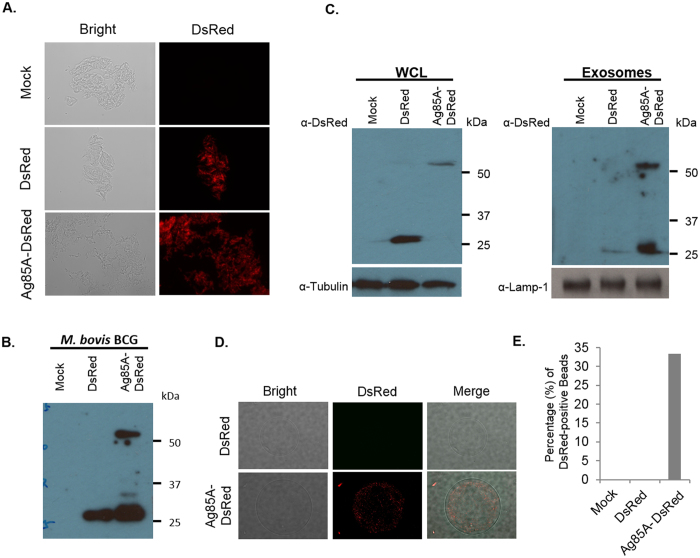
Increased trafficking of DsRed to exosomes in RAW264.7 cells infected with *M. bovis* BCG expressing the Ag85A-DsRed fusion protein. **(A)** Fluorescence microscopic and **(B)** western blot analysis of recombinant *M. bovis* BCG expressing DsRed or Ag85A-DsRed. **(C)** Western blot analysis of exosomes and RAW264.7 whole cell lysate (WCL) following a 72 hour infection with the recombinant *M. bovis* strains or from unifected cells. Tubulin and Lamp-1 were used as loading control for WCL and exosomes respectively. **(D)** Fluorescence microscopic analysis of Ds-Red positive exosomes captured on protein G Sepharose beads coupled to anti-CD63. **(E)** Quantitation of the DsRed-positive Sepharose beads observed in **(D)**. Uncropped western blots are shown in Supplementary Figure 1.

The interpretation of the Rab27a mouse experiments is complicated by Rab27a’s described role in other immune system functions^16^. Therefore, to further evaluate exosomes potential function in stimulating an antigen-specific T cell response during an *in vivo Mtb* infection, we generated a recombinant *M. bovis* BCG strains that expresses an Ag85A-DsRed fusion protein (Fig. 4A,B). Ag85A is a crucial component of the antigen 85 enzyme complex that functions as an acyltransferases and converts TMM (Trehalose monomycolate) to TDM (Trehalose dimycolate), and was previously identified in exosomes isolated from mycobacteria-infected macrophages^4^. In the recombinant BCG strain, Ag85A served as an exosome-targeting signal to facilitate DsRed trafficking to exosomes. *M. bovis* BCG strain expressing DsRed alone was also generated for this study. Both strains showed indistinguishable growth curves. As presented in Fig. 4 both the DsRed and Ag85A-DsRed were expressed by the recombinant *M. bovis* BCG. Moreover, both proteins were present in the macrophage cell lysate following infection with the recombinant *M. bovis* BCG (Fig. 4C). However, the DsRed was present in exosomes at significantly higher concentration in cells infected with BCG expressing the Ag85A-DsRed fusion protein compared to cells infected with BCG expressing DsRed alone. Interestingly there appeared to be some cleavage of the linker between Ag85A and DsRed within the exosome as we observed both intact fusion protein as well as DsRed alone in the exosomes. Exosomes derived from mammalian cells may be discriminated from other types of extracellular vesicles using various surface biomarkers including CD63 and CD9^17^. To confirm that the DsRed-containing vesicles were exosomes, CD63-positive vesicles were captured on Protein G-conjugated sepharose resins and then analyzed by fluorescence microscope. As shown in Fig. 4D,E, CD63 positive extracellular vesicles released from macrophages infected with *M. bovis* BCG expressing Ag85A-DsRed were positive for DsRed fluorescence. No detectable fluorescence was observed for vesicles released from macrophages infected with *M. bovis* BCG expressing DsRed alone. Altogether the data indicates that Ag85A can be used to significantly increase transport of DsRed to exosomes. Therefore *M. bovis* BCG expressing either Ag85A-DsRed or DsRed provides a new tool to better address the importance of exosomes in driving a T cell response during an *Mtb* infection.

### Exosomes contribute to a DsRed specific T-cell response in mice infected with *M. bovis* BCG expressing Ag85A-DsRed

**Figure 5:**
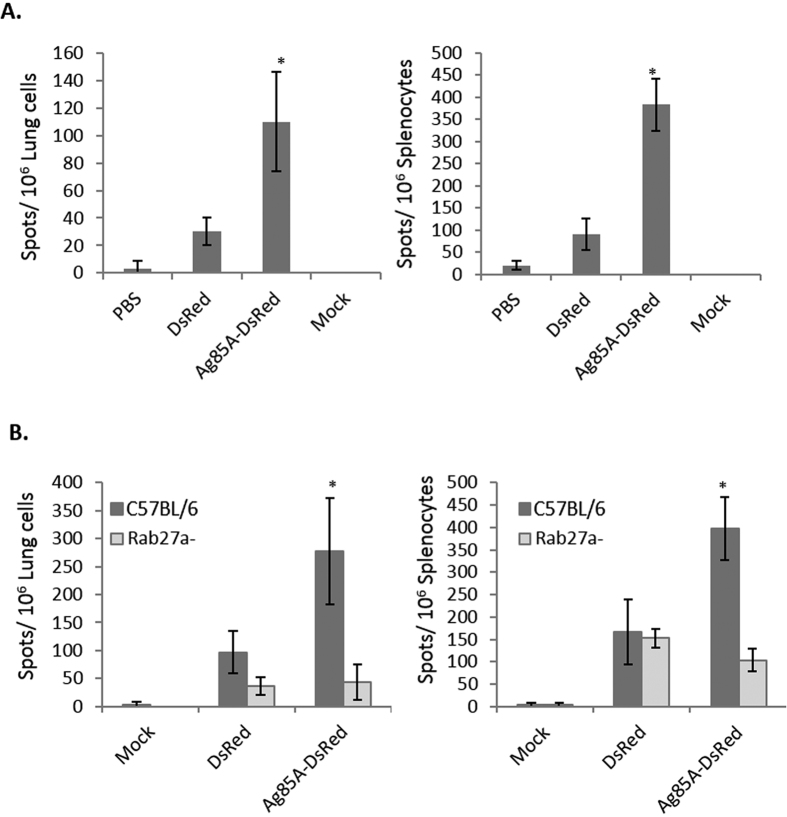
DsRed-specific T cell response is elevated in mice infected with *M. bovis* BCG expressing Ag85A-DsRed compared to bacilli expressing DsRed only. **(A)** Wild-type mice were intranasally infected with *M. bovis* BCG expressing DsRed or Ag85A-DsRed and the lung and splenic cells were harvested 2 weeks post-infection and stimulated *ex-vivo* with DsRed. The number of IFN-ɣ producing T cells responding to the DsRed antigen was measured by ELISPOT. **(B)** Wild-type C57BL/6 or Rab27a-deficient mice were intranasally infected with *M. bovis* BCG expressing either DsRed or Ag85A-DsRed. The number of IFN-ɣ producing T cells responding to the antigen DsRed was determined by ELISPOT. Results are defined for each mouse (4 mice/group) +/−SD and statistical analysis was performed between BCG expressing DsRed or Ag85A-DsRed **(A)** or between infected WT and Rab27-deficient mice **(B)** (*p < 0.05). The data is representative of two independent experiments.

Wild-type C57BL/6 mice were intranasally infected with the *M. bovis* BCG strains expressing DsRed or Ag85A-DsRed and the number of DsRed-specific T cells that produce IFN-ɣ was measured by ELISPOT. As shown in Fig. 5A, spleen and lung cells isolated from mice following a two week infection with *M. bovis* BCG expressing Ag85A-DsRed contained a significantly higher number of IFN-ɣ positive T cells after *ex vivo* DsRed stimulation compared to cells isolated from mice infected with DsRed-expressing BCG. To exclude the effect of other antigen-delivering pathways, Rab27a-deficient mice were used. Analogous to our results with the *Mtb* antigens, the absence of functional Rab27a impaired the delivery of Ag85A-DsRed to exosomes. The abundance of Ag85A-DsRed in exosomes isolated from Rab27a deficiency BMMs was 5-fold lower than observed in exosomes from wild type BMMs (Supplementary Figure 6). Therefore we hypothesized that Rab27a-deficient mice would show a limited T cell response to DsRed even when infected with *M. bovis* BCG expressing Ag85A-DsRed. As predicted, spleen and lung cells isolated from Rab27a-deficient mice infected with Ag85A-DsRed expressing *M. bovis* BCG showed a reduced number of T cells producing IFN-ɣ upon DsRed stimulation when compared to similarly infected wild-type mice (Fig. 5B). Further, we observed no significant difference in the number of IFN-ɣ positive T cells following *ex vivo* DsRed stimulation when we used spleen or lung cells isolated from Rab27a-deficient mice that were infected with recombinant *M. bovis* BCG expressing either Ag85A-DsRed or DsRed alone (Fig. 5B). These results show that host-derived exosomes can function in promoting an antigen specific T cell response during a *M. bovis* BCG mouse infection.

### Targeting HspX to exosomes during an M.tb mouse infection promotes the T cell response to this mycobacterial antigen

**Figure 6:**
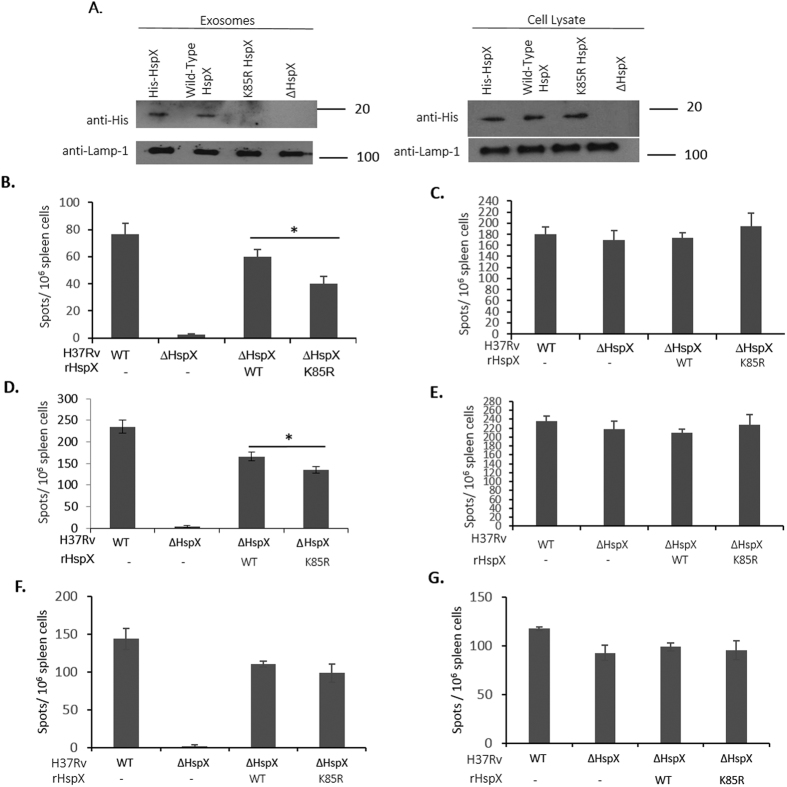
Increased trafficked of HspX to exosomes during a *Mtb* mouse infection enhances an HspX-specific T cell response. **(A)** RAW264.7 cells were infected with ∆HspX H37Rv or ∆HspX H37Rv expressing either His-tagged wild type HspX or K85R HspX and 72 hours post-infection exosomes were purified from the culture media. Raw264.7 cell lysates were also obtained. A separate well of Raw264.7 cells were treated with 40 μg/ml of purified His-tagged HspX (rHspX) and 24 hours post-treatment culture media and cell lysates were obtained. 10 μg of purified exosomes or cell lysates was analyzed for the His-tagged HspX using an anti-His antibody. Blots were also probed for Lamp1 as a loading control. (**B–E**) Splenocytes were harvested from wild-type C57BL/6 mice 10 days (**B,C**) or 15 days (**D**,**E**) post infection or from Rab27a-deficient mice 10 days post-infection (**F**,**G**). Mice (3 to 4 mice/group) were infected with either 10^6^ CFU of wild-type H37Rv, ∆HspX H37Rv or ∆HspX H37Rv expressing either wild-type HspX or K85R HspX. The splenocytes were analyzed 20 h after *ex vivo* stimulation with 5 μg/ml HspX (**B**,**D**,**F**) or 10 μg/ml *Mtb* CFP (**C**,**E**,**G**). The number of T cells producing IFN-ɣ upon HspX antigen stimulation was determined by ELISPOT and the number of positive cells counted +/−SD between individual mouse infections. Significance between samples is indicated (*p < 0.05). Data is representative of three independent experiments. Uncropped western blots are shown in Supplementary Figure 1.

While the BCG experiments indicate that exosomes can promote an antigen-specific T-cell response, we sought to develop a model to evaluate whether exosomes can promote T-cell activation to a mycobacterial protein during an *Mtb* infection. Previous studies have shown that the mycobacterial protein heat shock protein X (HspX) is present in exosomes isolated from human TB patient serum^18^. Recent studies have shown that trafficking HspX to exosomes was dependent upon mono-ubiquitination of a lysine residue at position 85. Therefore, we expressed in *M. smegmatis* an HspX protein with a Lysine to Arginine amino acid substitution at position 85 as well as wild-type HspX. Although expressed in *M. smegmatis* at similar levels to wild-type HspX, the lysine mutant was not trafficked to exosomes^19^. Therefore, we transformed into an ΔHspX strain of H37Rv, expression clones containing the HspX gene coding for either wild-type or the K85R mutant protein. Similar to our observation with *M. smegmatis*, wild-type HspX but not the K85R mutant was trafficked to exosomes in macrophages infected with the transformed ΔHspX H37Rv (Fig. 6A). Importantly both wild-type and K85R HspX were expressed and could be detected within the infected macrophage cell lysates. As a control, macrophages were treated with recombinant His-tagged HspX and as observed previously^19^, the HspX was endocytosed and trafficked to exosomes (Fig. 6A). Since our experiments demonstrated that K85R HspX is expressed by the transformed H37Rv but not targeted to exosomes we retro-orbitally infected C57BL/6 mice with the various H37Rv strains and the number of splenic T-cells which produced IFN-ɣ upon *ex vivo* stimulation with purified HspX was measured by ELISPOT. Splenocytes isolated 10-days post-infection with the H37Rv expressing the K85R HspX showed a significantly reduced number of IFN-γ producing T-cells (~35% decrease) compared to splenocytes isolated from mice infected with H37Rv expressing wild-type HspX (Fig. 6B). At 15 days post-infection the difference in the T-cell IFN-γ recall response was limited to ~15% but was still statistical significant (Fig. 6D). However, by day 21 post-infection there was no statistical difference in IFN-γ production between HspX-stimulated splenocytes isolated from mice infected with *Mtb* expressing either wild-type or K85R HspX. To ensure that differences in the observed T-cell immune responses were not due to differences in the *Mtb* strains, splenocytes were isolated 10 or 15 days post-infection and stimulated *ex vivo* with *Mtb* whole cell lysate and the number of IFN-γ producing cells quantified. As shown in Fig. 6C and E all the splenocyte populations isolated from mice infected with the different H37Rv strains showed a similar response when exposed *ex vivo* to a mixed population of *Mtb* antigens. Moreover, in contrast to wild-type C57BL/6 mice, we detected no statistical difference in IFN-γ production by splenocytes following *ex-vivo* stimulation with HspX or *Mtb* whole cell lysate when we used Rab27a-deficient mice for the 10 day infection (Fig. 6F,G). Our findings were not limited to the spleen as lung cells isolated from mice infected intratracheally with H37Rv expressing the K85R HspX showed a significantly reduced number of IFN-γ producing T-cells (~33% decrease) compared HspX-stimulated lung cells isolated from mice infected with H37Rv expressing wild-type HspX (Supplementary Figure 7).

## Discussion

Although there are approximately 2 billion people infected with *Mtb,* less than 10% of those infected will develop active disease in their life-time. This implies that in most individuals the immune response is sufficient to mediate protection. To understand this protective immune response and how it potential fails, requires a thorough knowledge of how PAMPs and *Mtb* antigens intersect with immune cells. Previous studies have demonstrated that exosomes released from infected macrophages are functionally associated with both the innate and acquired immune system. Exosomes are vesicles of 30–150 nm in size that are secreted from most nucleated cells and have been shown to function in intracellular communication. In the context of *Mtb* infection, we have identified a significant number of mycobacterial proteins on exosomes^4^,^20^. We have also shown that exosomes are immunogenic^21^,^22^. Exosomes derived from *M. bovis* BCG or *Mtb* infected macrophages upon adaptive transfer can activate antigen-specific CD4+ and CD8+ T cells *in vivo* and promote maturation of bone-marrow derived dendritic cells *ex vivo*^21^. Furthermore, we have shown that exosomes containing TB antigens can be used as an effective vaccine against an aerosolized *Mtb* infection in a mouse model^3^. While this data suggests exosomes can stimulate a T-cell immune response, there are several other processes through which antigen-specific T-cells may be primed including necrotic cells, apoptotic bodies, and free soluble antigen^2^,^23^,^24^. In the present study we developed new tools through which we could address the role of exosomes in stimulating innate and acquired immune responses during a natural infection.

One of the challenges in defining exosome function during the course of a natural infection is the lack of a mouse model in which exosome biogenesis is specifically inhibited. This is, in part, due to the fact that much of the machinery required for exosome biogenesis, such as the endosomal sorting complexes required for transport (ESCRT) machinery, mediates other important cellular processes and the loss of such machinery is embryonic lethal or has off target effects on cell/organ function. In the present study, we use a Rab27a-deficient mouse strain that was generated through a spontaneous mutation in the parental C3H strain and was back-crossed into the C57BL/6 background^25^. Rab27a, a member of the Rab GTPases, is known to mediate MVB fusion to the plasma membrane during exosome secretion^10^, although this may be cell-type specific. To evaluate Rab27a in the context of exosome biogenesis in macrophages, we compared exosome production by Rab27a deficient and wild-type bone marrow-derived macrophages and found significantly lower exosome concentration in the culture media of Rab27a-deficient macrophages. Interestingly, we observed an even greater difference in exosome concentration between wild-type and Rab27-deficient macrophages following an *Mtb* infection. This was due to an elevated concentration of exosomes released upon infection of wild-type macrophages while no significant increase was observed for infected Rab27a-deficient macrophages. Similar results were observed *in vivo* as serum concentration of exosomes increased only ~5 fold after a 40 day *Mtb* infection of Rab27a-deficient mice compared to an ~15 fold increase for wild-type mice infected with *Mtb*. It is likely that the decreased exosome concentration observed in the serum of infected Rab27a-deficient mice is due to combined decrease in exosomes released from infected macrophages as well as from other cell types whose exosome release is dependent on Rab27a. However, which cell types are producing exosomes during the course of an *Mtb* infection awaits further investigation.

In addition to decrease exosome production, the population of exosomes secreted from *Mtb* infected Rab27a-deficienct macrophages showed a reduced level of mycobacterial proteins and reduced capacity to stimulate TNF-alpha and RANTES production. Analysis of host exosomal markers showed that the exosomes secreted from the Rab27a deficient macrophages were CD81+ but CD63-, in part, reflecting an early study by Bobrie *et al*. which demonstrated Rab27a deficiency results in the secretion of exosomes that have altered host protein composition, most notably reduced CD63 expression^26^. At present, it is unclear whether the decreased expression of mycobacterial proteins in Rab27a-deficient exosomes reflects the inability of the macrophage to effectively secrete exosomes, or rather represents a distinct subpopulation of exosomes generated through a unique biogenesis and protein sorting pathway.

Altogether our *in vivo* and *in vitro* data using the Rab27a deficient macrophages and mice suggest a role for this GTPase in activation of both the innate and acquired immune response following an *Mtb* infection. This decreased immune response correlates with diminished release of exosomes and limited transport of mycobacterial proteins to exosomes. This suggest that the effects of Rab27a deficiency on the immune response to *Mtb* stems in part from its effect on exosome production/composition. However, while it is known that Rab27a plays a role in exosome biogenesis, Rab27a deficiency is also associated with immune system dysregulation^16^. For example, it is known that Rab27a mediates exocytosis in neutrophils, CTLs, NK cells and mast cells, which in the context of immunity, is important for the secretion of antimicrobials. Rab27a associated defects in degranulation may lead to defects of adhesion, migration, chemotaxis of cells and defects in neutrophil mediated phagocytosis and reactive oxygen species production. However, characterization of the CD4+ T-cell responses in Rab27a- deficient mice have shown that the deficiency does not affect CD4+ T-cell proliferation and maturation and that T-cell receptor mediated IFN-y production is normal^27^. We also observed no difference in the number of T cells present in the spleens of Rab27a-deficient and wild-type mice prior to infection (data not shown). Therefore, we believe the model and the initial data establishes exosomes as a potential mediator of T-cell activation.

Nevertheless, as an alternative approach to evaluate exosomes in promoting T cell activation we developed a recombinant *M. bovis* BCG strain in which the fluorescent reporter protein DsRed was expressed either alone or fused to Ag85A. In this context, we used Ag85A as a protein to target DsRed to exosomes as we have previously shown Ag85A to be a major constituent of exosomes isolated from infected macrophages, mice and human TB patients^4^,^18^,^20^. Our BCG infection experiments indicate that targeting DsRed to exosomes enhanced the T cell response to this protein antigen. However, T-cell activation may also be mediated through other mechanisms such as free-soluble antigen or apoptotic blebs. Furthermore, a more recent study by Athman *et al*. suggests that bacterial membrane vesicles secreted directly from *Mtb* function to disseminate mycobacterial proteins which can subsequently interact with the immune system^28^. In order to exclude these other mechanisms of T-cell activation against DsRed, we applied our *M. bovis* BCG expressing Ag85A-DsRed or DsRed alone to our Rab27a-deficient mouse which as indicated previously has a markedly diminished exosome production relative to wild-type macrophages or mice. The number of DsRed-specific T cells was comparable in Rab27a-deficient mice whether they were infected with recombinant *M. bovis* BCG expressing Ag85A-DsRed or expressing DsRed alone. To address exosomes as carriers of antigen for T cell activation more directly and in the context of an *Mtb* infection, we generated an H37Rv strain that expressed either WT HspX or a mutant HspX which is not trafficked to exosomes upon macrophage infection^19^. When mice were infected with *Mtb* expressing WT HspX we observed a significantly higher number of IFN-ɣ positive T cells specific to HspX when compared to mice infected with *Mtb* expressing the K85R HspX. Interestingly, this exosome mediated T cell activation may be more relevant during the initial T cell response as we observed a diminished role for exosomes at 15 and 21 days post-infection relative to 10 days. Moreover, as observed with the BCG expressing Ag85a-DsRed, we again detected no significant difference in the production of IFN-γ by splenocytes isolated from Rab27a-deficient mice infected with H37Rv expressing either WT or K85R HspX, further suggesting that exosomes are driving a T cell response during a natural *Mtb* infection.

Although our data indicates that exosomes produced during a mouse *Mtb* infection can promote a T cell response, this is clearly only one of a number of mechanisms by which antigens can be made available for presentation to T cells. Recent studies by Srivastava *et al*. suggest that blocking release of non-vesicular “free” antigen from infected macrophages promotes antigen presentation by infected DC both *in vitro* and *in vivo*^9^. The authors suggest that releasing antigen out of infected DCs and macrophages is a virulence mechanism used by *Mtb* to limit T cell activation. However, it is unclear from these experiments how much of *Mtb* antigens are presented through cross-priming compared to presentation by infected cells during a natural infection. Our data shows that cross-presentation through exosomes occurs during an *Mtb* infection and our Rab27a data suggest that limiting exosome-mediated immune response leads to higher bacterial burden. Since greater than 90% of individuals infected with *Mtb* mount a T cell response sufficient to control the infection and the established observation that infected DCs/macrophages are poor presenters of antigen, it suggest that the various mechanisms of antigen cross-presentation including exosomes are required for the protective adaptive immune response. It is also possible that individual variations in exosome-mediated or other mechanisms of antigen presentation can be an important factor in those individuals who succumb to TB.

In summary, our data indicates that exosomes can mediate immune system activation during an *in vivo* infection. Furthermore, we demonstrate that exosomes enhance T-cell activation during an *Mtb* infection. However, the importance of exosome-mediated antigen delivery compared to other mechanisms of antigen presentation requires additional study and may vary depending on the stage or route of infection as well as on which antigen is being evaluated and its distribution inside the infected host cell.

## Methods

### Ethics Statement

The University of Notre Dame is accredited through the Animal Welfare Assurance (#A3093-01) and follows the guidelines described in “*The Guide for the Care and Use of Laboratory Animals*” and the “*United States Department of Agriculture Animal Welfare Act and Animal Welfare Regulations*”. All animal procedures were approved by the Institutional Animal Care and Use Committee (approval # 14-08-1969).

### Animals

All wild type C57BL/6 mice and Rab27a-deficient mice in a C57Bl/6 background were housed at the institutional animal facility under specific-pathogen-free conditions during the experiments. The Rab27a-deficient mice were generously provide by Dr. Sergio Catz, Scripps Research Institute, CA. *M.* bovis BCG infections were carried out in the biosafety level-2 laboratory and *Mtb* infections were performed in a biosafety level-3 laboratory.

### Bacteria and and Macrophage Cell Lines

*M. bovis* BCG and *M. tuberculosis* H37Rv strains were grown in Middlebrook 7H9 broth medium (Difco, Becton-Dickinson) containing 10% OADC (oleic acid/albumin/dextrose/catalase, 0.05% Tween 80) until exponential phase and then aliquoted and stored at −70 °C until use. Prior to use, the bacterial stocks were thawed and the mycobacteria were de-clumped by a brief sonication and passed through a syringe fitted with a 27-gauge needle at least 10 times. The macrophage cell line RAW 264.7 (ATCC, Manassas, VA) was maintained in Dulbecco modified Eagle’s minimal essential medium (DMEM, Cellgro, Manassas, VA) supplemented with 10% fetal bovine serum (Hyclone, South Logan, Utah), 25 mM Na-HEPES (ThermoScientific, Rockford, IL), 1 mM Sodium pyruvate (Lonza, Walkersville, MD), 100 U/mL penicillin and 100 U/mL streptomycin (Hyclone, South Logan, Utah) at 37 °C with 5% CO_2_. Bone marrow-derived macrophages (BMMs) were prepared from wild type C57BL/6 or Rab27a deficiency mice as described previously^29^.

### Construction of Plasmids

To generate plasmid pMV261-Ag85A::DsRed, the DNA fragment encoding DsRed was amplified by PCR from plasmid, pMSP12-DsRed2, using Easy-A high-fidelity DNA polymerase (Agilent Technologies, CA) and primers DsRed-Forward (NheI), 5′-CTT GCT AGC ATG GCC TCC TCC GAG AAC GT-3′, and DsRed-Reverse (Sna BI), 5′-CTT TAC GTA CTA CAG GAA CAG GTG GTG GC-3′. Ag85A ORF was amplified from *M. tuberculosis* genomic DNA with the primers Ag85A-Forward (BamHI), 5′-CTT GGA TCC ATG CAG CTT GTT GAC AGG GTT CG-3′, and Ag85A-Reverse (NheI), 5′-CTT GCT AGC AGG TCC GGC GCC CTG GGG CGC G-3′. The PCR products were cloned into pGEM-T easy vector to generate plasmids pGEM-DsRed and pGEM-Ag85A, respectively. The DsRed fragment was cut from pGEM-DsRed with restriction endonucleases NheI and NdeI and inserted into plasmid pGEM-Ag85A at the same sites to create plasmid, pGEM- Ag85A::DsRed. Finally, the Ag85A::DsRed fragment was cut from pGEM- Ag85A::DsRed with restriction endonucleases BamHI and SnaI and cloned in the vector pMV261.hgy at the sites BamHI and HpaI to generate plasmid pMV261-Ag85A::DsRed. To generate plasmid pMV261-DsRed, the ORF of DsRed was amplified from plasmid,pMSP12-DsRed2 with primers DsRed-Forward (BamHI), 5′-CTT GG ATCC ATG GCC TCC TCC GAG AAC GT-3′, and DsRed-Reverse (Sna BI), 5′-CTT TAC GTA CTA CAG GAA CAG GTG GTG GC-3′. The PCR product was cloned into pGEM-T Easy vector to create plasmid pGEM-DsRed, and then the DsRed fragment was cut from pGEM-DsRed with restriction endonucleases BamHI and SnaI and cloned into the vector pMV261.hgy at the sites BamHI and HpaI to generate plasmid pMV261-DsRed.

The pMV261-HspX and pMV261-HspX mutant plasmids construction was generated as previously described^19^.

### Electroporation of Mycobacteria

*M. bovis* BCG and *Mtb* were grown in Middlebrook 7H9 broth plus 10% OADC at 37 °C until OD600 at a range of 0.5–1.0. The cells (50 ml culture) were washed with 50 ml 10% glycerol three times at 2000 × g, 15 min, RT and cell pellet was finally resuspended in 500 μl 10% glycerol. For transformation, 200 μl competent mycobacteria were gently mixed with 2 μg of plasmid DNA for BCG or with 1 μg of plasmid DNA for *Mtb* and then incubated in ice 30 min. The DNA-Cell mixture was transferred into a 0.2 cm electroporation cuvette and pulsed with BioRad GenePulser Xcell™ Electroporation Systems set to 2.5 kV, 1000 ohms and 25 μF. The cells were then transferred into a 15 ml snap cap tube containing 5 ml Middlebrook 7H9 broth containing 10% OADC and incubated at 37 °C over-night. The transformation was than plated on Middlebrook 7H10 agar plates containing 10% OADC and 50 ug/ml hygromycin and incubated at 37 °C for 3–4 weeks.

### Exosome Preparation and Purification

RAW 264.7 cells or BMMs were infected with *M. bovis* BCG strains or *Mtb* as described previously^15^. After 72 hours in exosome-depleted medium, cell culture supernatant was collected and centrifuged at 350 × g for 15 min at 4 °C to remove cells and large debris. The supernatant was then passed through a 0.22 um polythersulfone filter (Corning, NY, USA), followed by centrifugation at 10,000 × g for 1 hour at 4 °C. The culture supernatant was further ultra-centrifuged at 100,000 × g for 1 h at 4 °C to spin down expected exosomes. The pellets were resuspended in PBS and washed 3X with PBS. The final pellets were resuspended in PBS and stored at −80 °C.

### NanoSight Analysis

The exosomes were characterized for size distribution and quantitated by NanoSight LM10, using light scatter from the 635-nm red laser, as well as NTA 2.3 Analytical software as described^30^.

### Cytokine Array and TNF-alpha, RANTES and IFN-ɣ ELISA

Exosomes were isolated from H37Rv-infected WT or Rab27a infected mice or PBS-injected control mice. Primary bone marrow macrophages (1 × 10^6^ cells) were stimulated with the serum-derived exosomes at 250 mg/ml for 16 hours. The cell culture supernatants were harvested, and particulates were removed by centrifugation. The supernatants were tested immediately for cytokine/chemokine levels, using the Mouse Cytokine Array Panel A kit (R&D Systems, Minneapolis, MN) according to the manufacturer’s instructions. Briefly, cell culture supernatants were mixed with a blend of biotinylated detection antibodies (Abs) and incubated with the nitrocellulose membrane that contains 40 different anti-cytokine/chemokine capture Abs spotted in duplicate. Any cytokine/Ab complex formed is bound to the immobilized capture Ab. The membranes were incubated with chemiluminescent substrate (Thermo Scientific [Pierce]. In separate experiments, the cell culture supernatants from the exosome treated macrophages were also tested for TNF-alpha by ELISA (Biosource) per the manufacturer’s instructions. RANTES levels in exosome treated macrophages was measured by ELISA per manufacturer’s instructions (R&D Systems, Camarillo, CA).

Wild type C57BL/6 mice and Rab27a-deficient mice were retro-orbitally infected with WT *Mtb* H37Rv, and cells from lungs and spleens of mice were harvested at various time points post-infection as described previously^3^. Isolated cells were stimulated *ex vivo* with 5 μg/ml *Mtb* whole cell lysate at 37 °C with 5% CO_2_. After 72 h, INF-γ released in cell-free culture supernatant was measured by ELISA (eBioScience, San Diego, CA) according to the manufacturer’s instruction.

### Histology of lung sections

The lung sections were fixed in 10% neutral buffered formalin overnight and then transferred to 70% ethyl alcohol. The samples were processed using a Shandon Citadell 2000 automated tissue processor. After fixation, samples were dehydrated, embedded in paraffin, and sectioned at 4 mm, using a Leica RM 2155 automated microtome. Sections were H&E stained to examine general tissue and cellular morphology. Individual sections were scored for pathology as described^3^.

### Western blots

Exosomes (10 μg or 10^9^ particles) were resuspended in PBS with protease inhibitors. The suspension was mixed with Laemmli buffer, heated at 95 °C for 5 min, and chilled on ice for 5 min before loading onto SDS gel. Immunoblots probed with antibodies for proteins: Tsg-101 (C-2, 1:1000, Santa Cruz), CFP (C192, 1:1000, ATCC), 19kDa-lipoprotein (IT-19, 1:1000, ATCC), His-tag (1:500, Santa Cruz), CD81 (1:500, SBI), CD63 (1:500, Systems Bioscience), and, DsRed (632393, 1:1000, Clontech). Primary antibody incubation was followed with HRP-conjugated secondary antibodies (1:25,000, Pierce) and detected using enhanced chemiluminecence kit (Pierce). As loading controls, primary mouse mAbs against either alpha-Tubulin, 1/1000 dilution (Cat. T9026, Sigma) or Lamp-1 1/500 dilution (SC-17768, Santa Cruz Biotechnology or 1D4B, DHSB) were used.

### Macrophage Infection and CFU determination

Mycobacteria were thawed and incubated with 10% horse serum for 2 hours at 37 °C to opsonize bacteria for infection. Rab27a or WT BMMs were seeded at 10^6^ cells/well in a 6-well plate. Bacteria were added to the cells at indicated concentrations and were allowed to infect for 4 hours at 37 °C. Cells were washed three times with phosphate-buffered saline (PBS) before fresh complete media without antibiotics was added. Macrophages were lysed immediately after the 4 hour infection or 76 hours post infection. Bacteria was plated on 7H11 agar and CFUs were determined after 4 weeks at 37 °C.

### Mouse Infection and CFU determination

C57BL/6 mice of Rab27a-deficient mice (8–10 weeks old) were infected retro-orbitally with H37Rv expressing wild-type or mutant HspX at (10^6^ bacilli per mouse) or injected with an equal volume of PBS. Mice were sacrificed at 10, 15 and 21 days post infection (three to four mice per group), and serum and spleens were collected. Exosomes were purified from mouse serum. Spleens from infected mice were homogenized and passed through a 70-mm cell strainer. The cell suspension was treated with RBC lysis buffer, followed by PBS washes. The suspension was serially diluted in PBS+ Tween 80 (0.05% v/v) and plated on 7H10 agar supplemented with oleic albumin dextrose catalase. CFUs were determined after 4 weeks at 37 °C.

For BCG infection, WT C57BL/6 mice and Rab27a-deficient mice (8–10 weeks old) were intranasally immunized with *M. bovis* BCG expressing DsRed or Ag85A-DsRed (1 × 10^6^ CFU/mouse) or PBS alone at an injection volume of 30 μl (15 μl/nostril) as described previously^30^. There were four mice per treatment group. Mouse lungs and spleens were isolated two weeks post-infection and used for ELISPOT analysis.

### Flow Cytometry

The cells were rinsed with Dulbecco’s PBS and gently scraped and counted on a hemacytometer, using trypan blue to assess viability. The cells were washed in FACS buffer and blocked with 10% mouse serum and stained with PE-conjugated anti-mouse CD69 (BD Pharmingen, San Diego, CA), FITC-conjugated CD4, or using isotype Abs as controls. Cells were analyzed for protein surface expression with a Beckman Coulter flow cytometer, and the percentage of positive cells was calculated.

### Isolation of Lymphocytes and ELISPOT

Mouse lung and spleen cells were isolated after an *Mtb, or M. bovis* BCG infection as described previously^3^. The IFN-ɣ assay was performed using the Mouse IFN-ɣ ELISPOT Ready-SET-Go! Kit (eBioScience, CA) following the manufacturer’s protocol. Spleen and lung cells (5 × 10^5^ cells/well) were analyzed 20 h after *ex vivo* re-stimulation with recombinant DsRed (STA-202, Cell Biolabs, INC) or HspX (Colorado State University) at a final concentration of 5 μg/ml or *M. tuberculosis* CFPs at 10 μg/ml.

### CD63-conjugated Bead-binding Assay

Protein G-Sepharose 4B resins (Cat. 10–1241, Invitrogen, CA) was first washed and incubated with blocking buffer (cat. 00-4202-56, ebioscience, CA) on a rotator at 4 °C for 5 hrs. Prepared resins were coupled with CD63 Abs (Cat. 15363, Santa Cruz, CA) at 4 °C overnight, followed by a wash step with blocking buffer. The resin was resuspended in blocking buffer and incubated with purified exosomes at 4 °C overnight. After washed with blocking buffer 3 times, the resins were analyzed by Zeiss AxioObserver. Z1 Fluorescence Microscope.

### Statistical Methods

The data obtained was analyzed by student paired T-test. A value of p ≤ 0.05 was considered significant. The computer program GraphPad PRISM 5 was used for the analysis.

## Additional Information

**How to cite this article:** Smith, V. L. *et al*. Exosomes function in antigen presentation during an *in vivo Mycobacterium tuberculosis* infection. *Sci. Rep.* **7**, 43578; doi: 10.1038/srep43578 (2017).

**Publisher's note:** Springer Nature remains neutral with regard to jurisdictional claims in published maps and institutional affiliations.

## Supplementary Material

Supplementary Information
