# Supplementary material for: Exosomes function in antigen presentation during an in vivo Mycobacterium tuberculosis infection
Source: Sci Rep. 2017 Mar 6;7:43578. doi: 10.1038/srep43578 (PMC5338015; doi:10.1038/srep43578)
Supplement: Supplementary Information [file srep43578-s1.pdf]

Exosomes function in antigen presentation during an *in vivo Mycobacterium tuberculosis* infection

Victoria L. Smith, Yong Cheng, Barry R. Bryant and Jeffrey S. Schorey*

Supplementary Figure 1: Unprocessed scans of original western blots used in the main figures

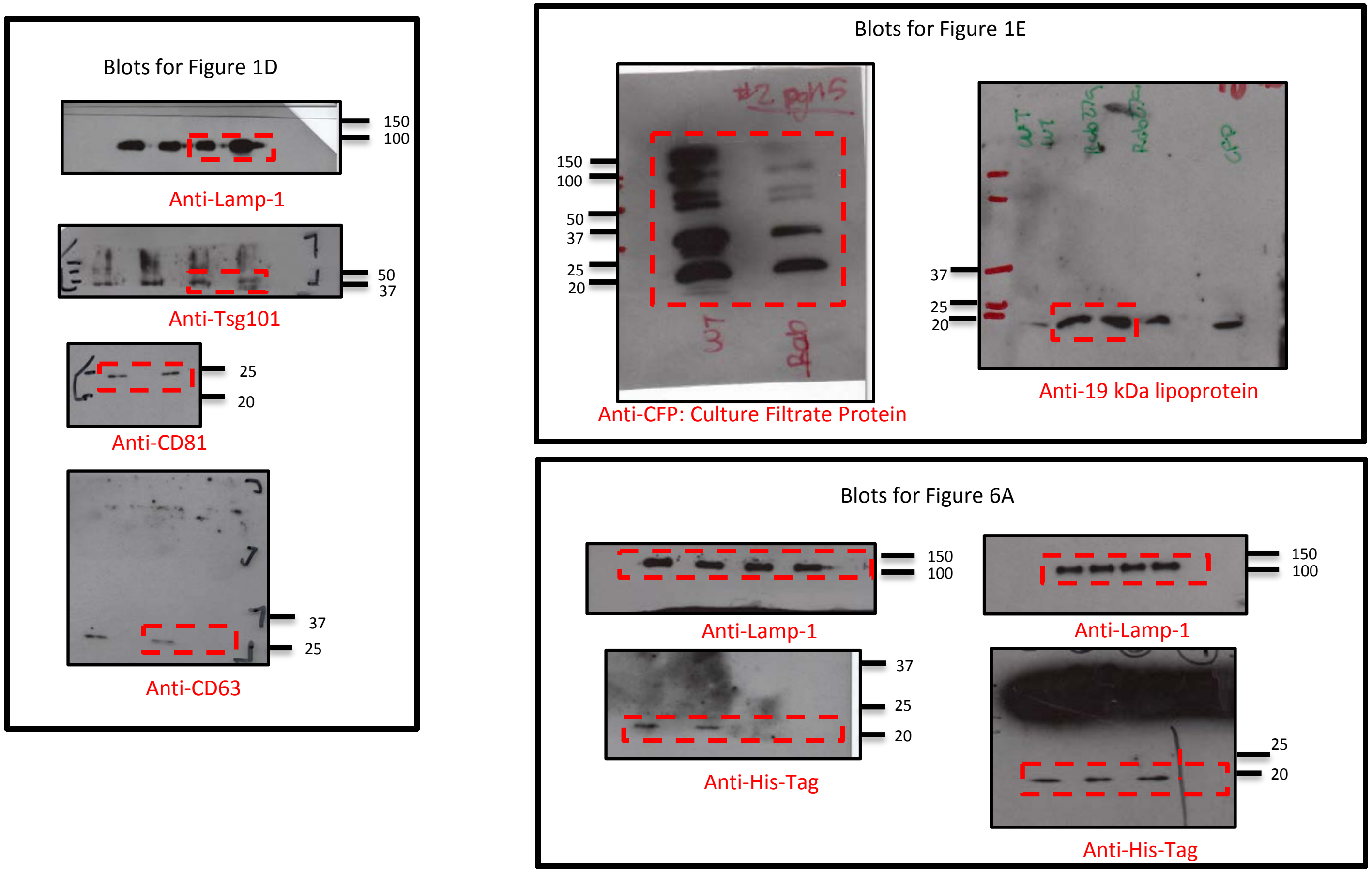

Uncropped full-length pictures of Western blot membranes. Membranes were often cut to enable blotting for multiple antibodies. Dashed lines indicate regions that were cropped.

Supplementary Figure 1 cont.

Blots for Figure 4B

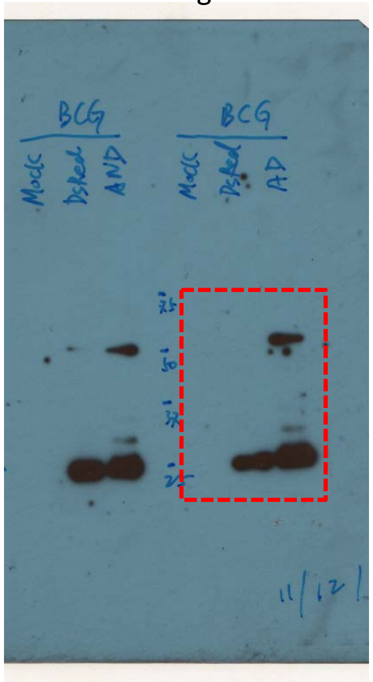

M. Bovis BCG
Whole Cell Lysate_ anti-DsRed

Blots for Figure 4C

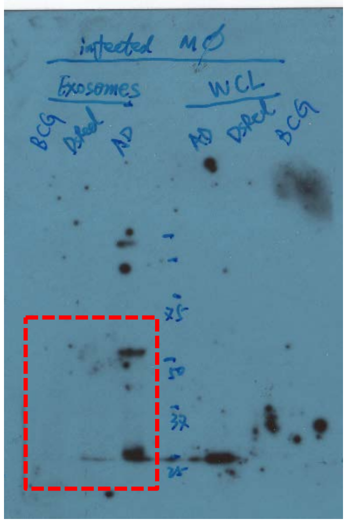

Exosomes_anti-DsRed

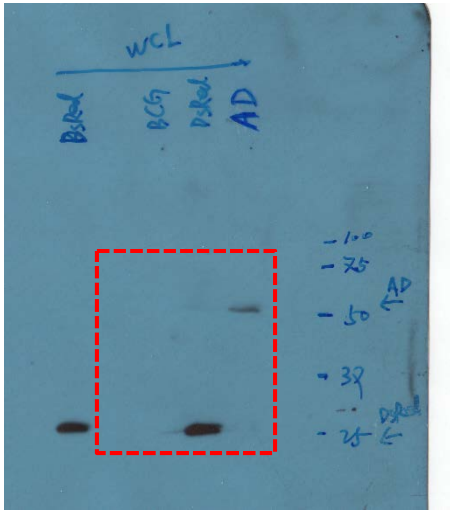

WCL_anti-DsRed

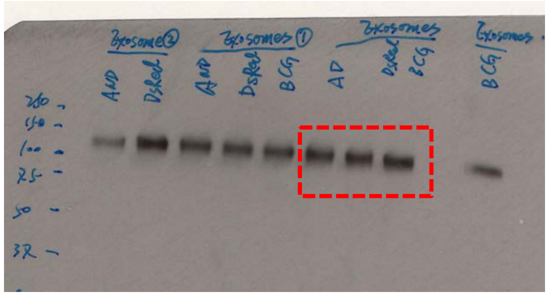

Exosomes_anti-LAMP1

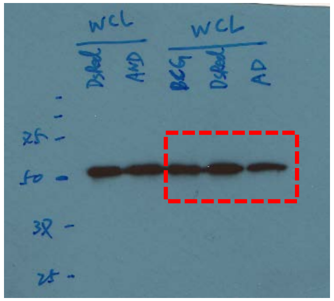

WCL_anti-Tubulin

Blots for Figure S6A

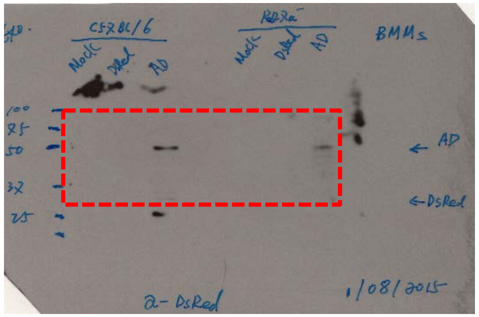

Exosomes_anti-DsRed

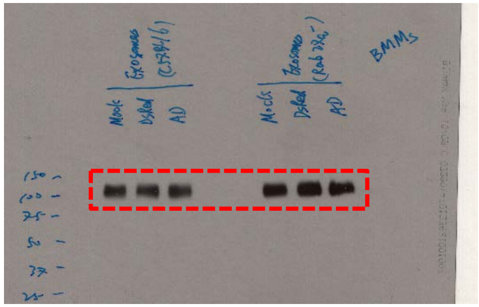

Exosomes_anti-LAMP1

Supplementary Figure 2

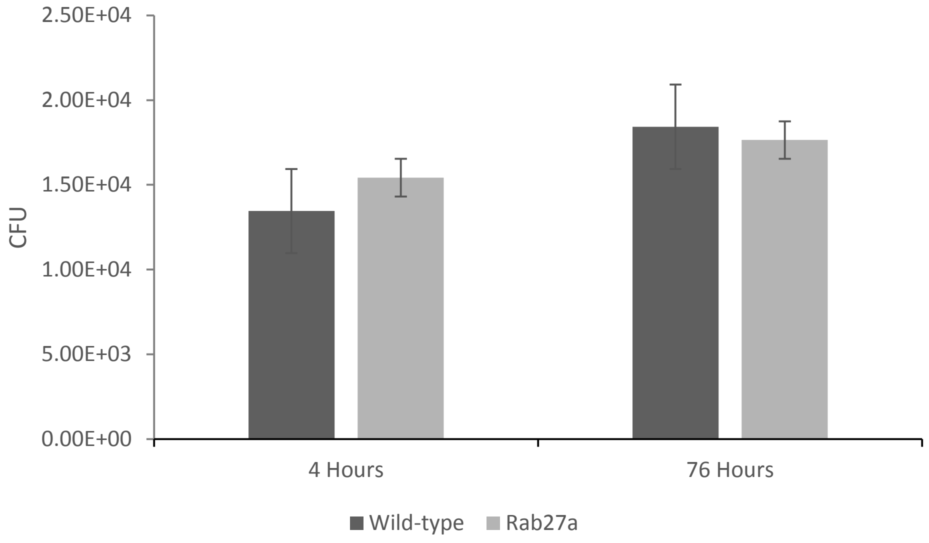

Supplementary Figure 2. Similar rates of infection and growth for *Mtb* in Rab27a-deficient and wild-type macrophages. Rab27a or wild-type bone marrow macrophages were seeded at 10^6 cells and infected with *Mtb* at a bacteria to macrophage ratio of 5:1. Macrophages were lysed at 4 hours and 76 hours post-infection. Bacteria was plated on 7H11 agar and CFUs were defined after 4 week incubation at 37^oC. Shown is the average CFU from three independent infections +/- SD.

Supplementary Figure 3

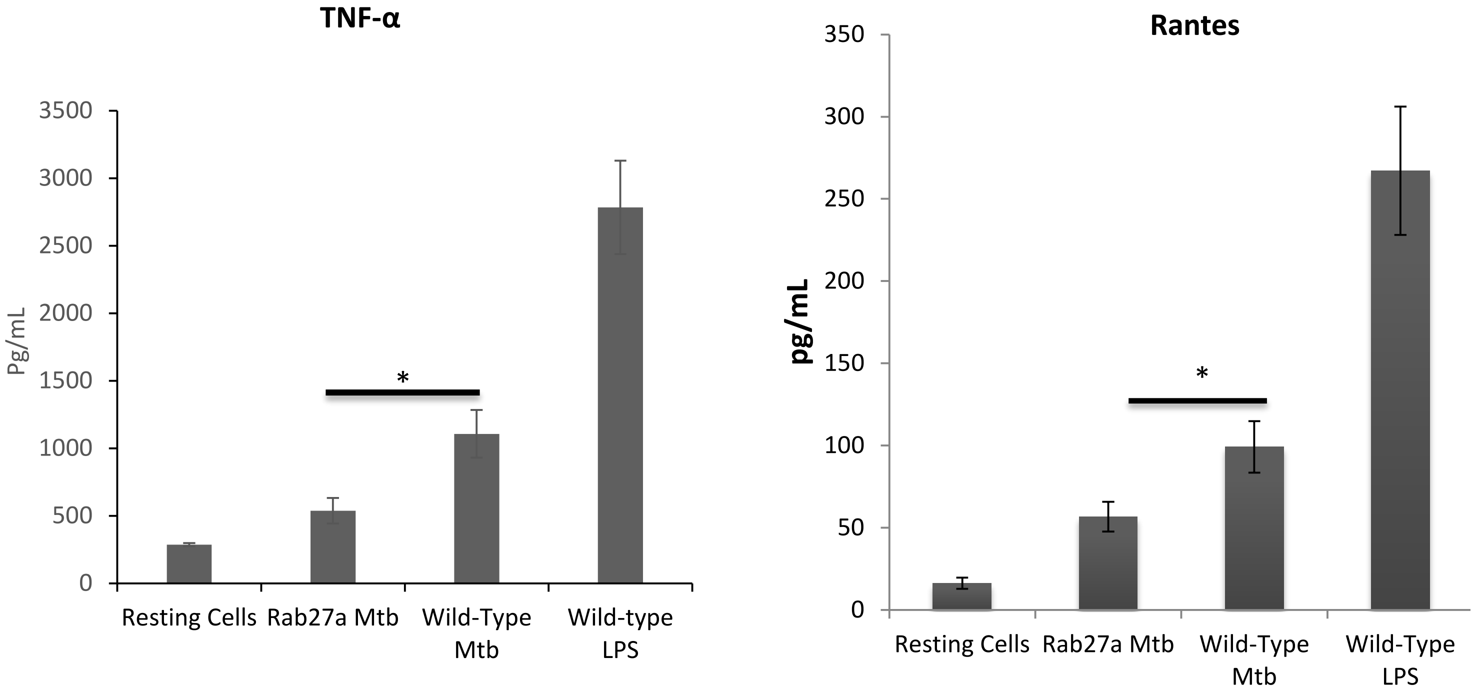

Supplementary Figure 3. Exosomes from *Mtb* infected Rab27a-deficient macrophages are less pro-inflammatory than wild-type derived exosomes. Exosomes were isolated from the cell culture supernatants of *Mtb* infected Rab27a-deficient or wild-type C57BL/6 BMMs. Naïve BMMs were treated with exosomes (40 μg/mL) for 16 hours and cell culture supernatant were collected and assayed for TNF-α and RANTES by ELISA. Data is the average of three independent infections +/- SD and representative of two independent experiments and statistical significance is indicated (*p<0.05).

Supplementary Figure 4

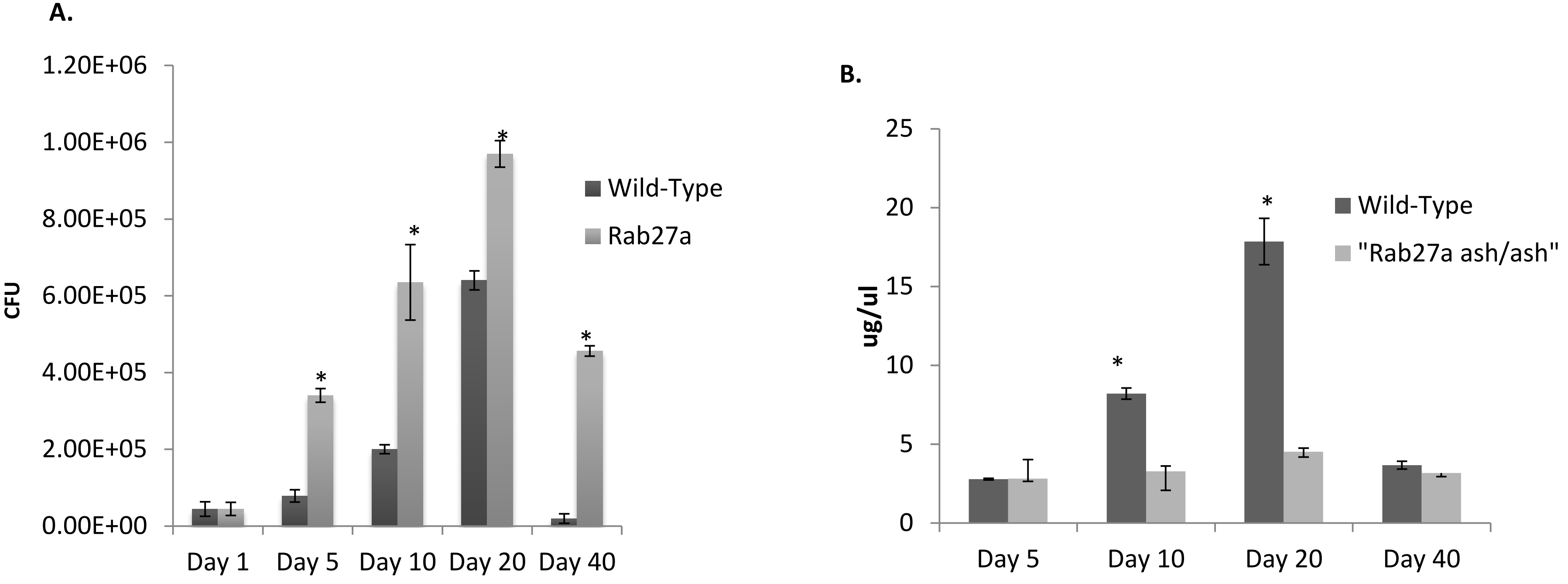

Supplementary Figure 4. Diminished exosome concentration and increased bacterial burden in Rab27-deficient mice compared to C57BL/6 mice following an *M. bovis* BCG infection**.** C57B/6 and Rab27a-deficient mice infected with 10^6 CFU *M. bovis* BCG by retro-orbital injection and were sacrificed at days 1, 5, 10, 20, and 40-post infection. Spleen CFU **(A)** serum exosome concentration **(B)** and were determined. Average CFU and exosome concentration from 3 to 5 mice per time point +/- SD. Significance between WT and Rab27a-deficient mice is indicated (*$p < 0.05$). Data is representative of two independent Experiments.

Supplementary Figure 5

A.

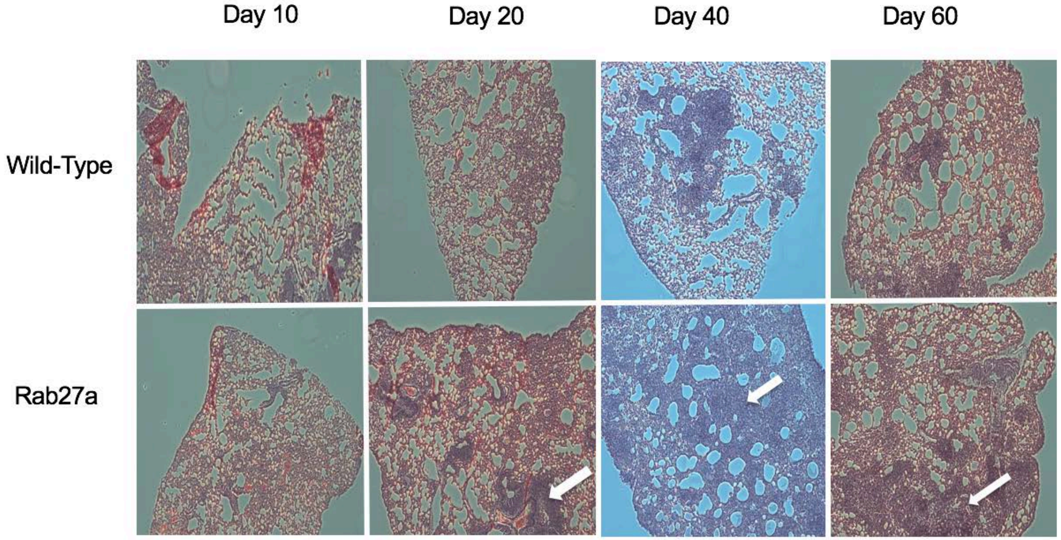

B.

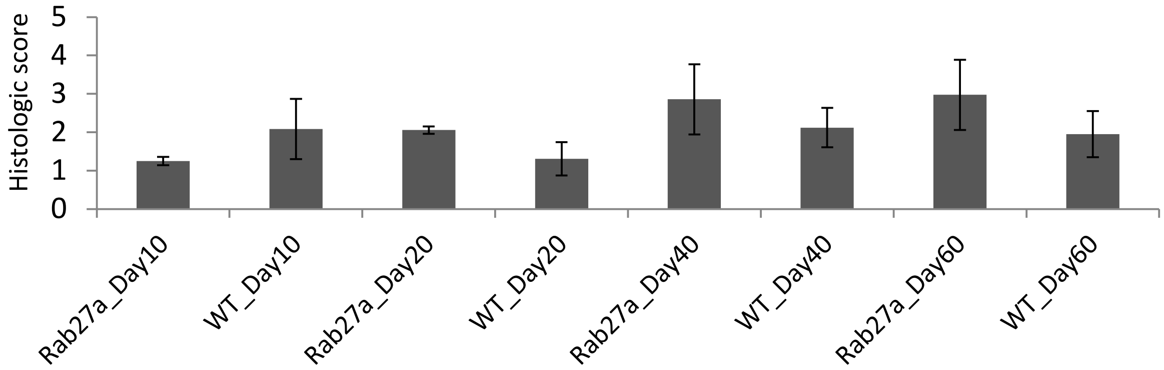

Supplementary Figure 5. Histology of lungs at different time's post-*Mtb* infection of WT or Rab27a-deficient C57BL/6 mice. H&E staining of lung sections following infection with virulent *Mtb* H37Rv. The lung sections were analyzed under low (50×) magnification and the granulomas indicated with white arrows. Pictures are representatives of lung sections from 4 mice per group and from one of three independent experiments **(B)**. Histopathological scores of lung sections. The scoring was performed on 6 to 9 sections for each of the 3 WT and Rab27a-deficient mice infected for the times indicated and the average score +/- SD for each condition is shown.

Supplementary Figure 6

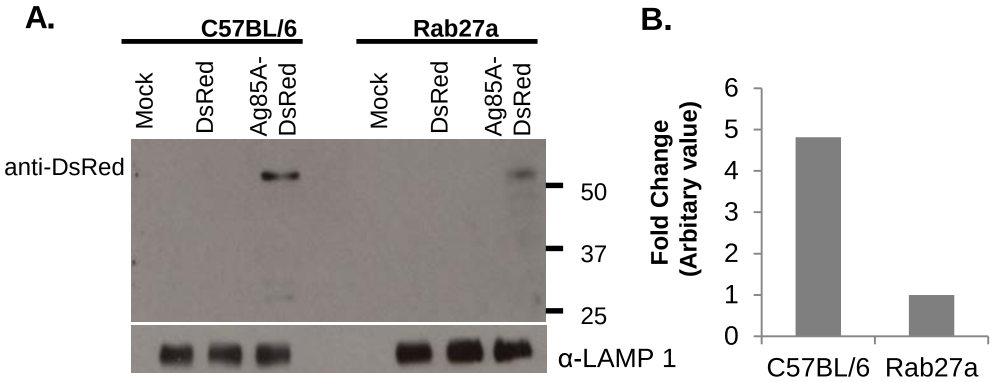

Supplementary Figure 6. Limited transport of DsRed to exosomes in Rab27a-deficient macrophages infected with Ag85A-DsRed expressing BCG. **(A)** Western blot analysis of exosomes isolated from the culture media of C57BL/6 or Rab27a-deficient BMMs infected for 72 hours with *M. bovis* BCG expressing Ag85A-DsRed. **(B)** Pixel intensity of the Ag85A-DsRed bands shown in (A).

Supplementary figure 7

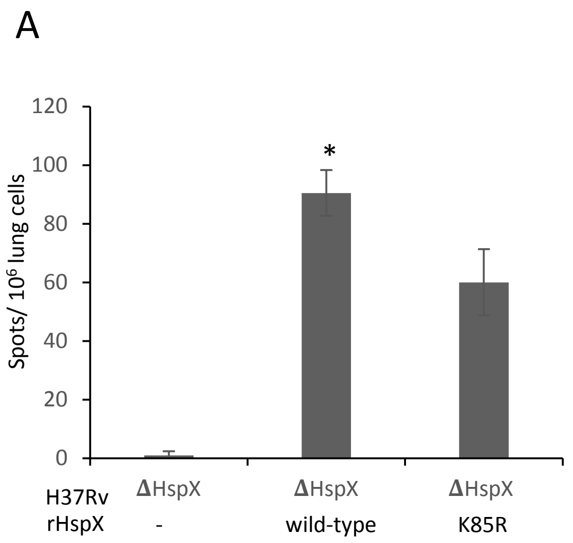

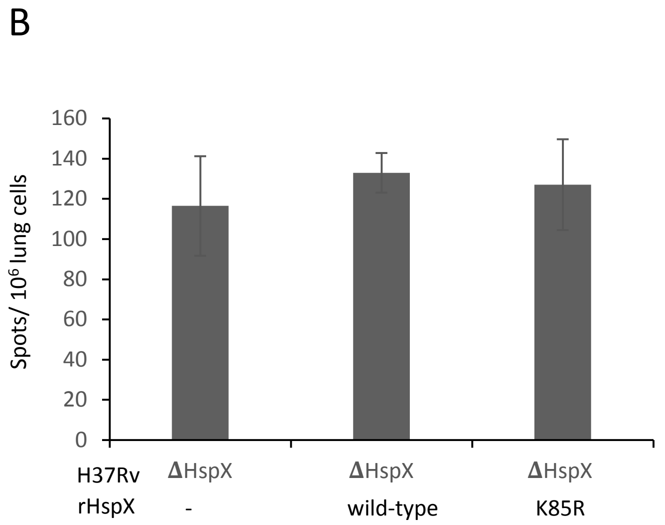

Supplementary figure 7. Lung cells were harvested from wild-type C57BL/6 mice 14 days post infection. Mice were infected with 10^5 ΔHspX H37Rv, or ΔHspX H37Rv expressing either his-tagged wild-type HspX or K85R HspX by intratracheal injection. The lung cells were analyzed 20h after *ex vivo* stimulation with 5 µg/ml HspX **(A)** or with 10 µg/ml of *Mtb* CFP **(B)**. ELISPOT for IFN-γ positive cells was performed and the number of positive cells quantified. Lung cells from individual mice (4 mice/group) were treated with antigen and the number of positive cells quantified +/-SD and statistical significance is indicated (*p<0.05).
